# The “black box” of treatment: Patients’ perspective on what works in opioid maintenance treatment for opioid dependence

**DOI:** 10.1186/s13011-021-00378-7

**Published:** 2021-05-10

**Authors:** Teresa C. Silva, Fredrik B. Andersson

**Affiliations:** 1 Department of Humanities and Social Sciences, Mid Sweden University, 10 – 85170 Holmgatan, Sundsvall, Sweden; 2 Risk and Crisis Research Center, Mid Sweden University, Kunskapens väg 1, Stapelmohrs väg, 831 40 Östersund, Sweden

**Keywords:** Opioid maintenance treatment, Opioid dependence, Substance use disorders, Patient perspective, Quality of life

## Abstract

**Background:**

A lack of conceptual modeling of how the components of opioid maintenance treatment (OMT) for opioid dependence (OD) work causes it to occasionally be labeled the “black-box” of treatment. This study had a two-fold objective: First, to analyze which factors related to OMT for OD contribute to the abstinence of problematic use of non-prescribed opioids and sustain recovery, from the patients’ perspective; second, to understand which changes OMT produced in the individuals’ lives might significantly contribute to relapse prevention.

**Methods:**

We used qualitative methods of design, inquiry, and analysis from a convenience sample of 19 individuals in a Swedish treatment setting.

**Results:**

All the participants reported previous cycles of problematic use of non-prescribed opioids and other non-prescribed psychoactive substances, treatment, abstinence, recovery, and relapse before starting the current OMT program. During the pre-treatment stage, specific events, internal processes, and social environments enhanced motivation toward abstinence and seeking treatment. During the treatment stage, participants perceived the quality of the human relationships established with primary social groups as important as medication and the individual plan of care in sustaining recovery. From the participants’ perspective, OMT was a turning point in their life course, allowing them a sense of self-fulfillment and the reconstruction of personal and social identity. However, they still struggled with the stigmatization produced by a society that values abstinence-oriented over medication-assisted treatments.

**Conclusion:**

OMT is not an isolated event in individuals’ lives but rather a process occurring within a specific social context. Structural factors and the sense of acceptance and belonging are essential in supporting the transformation. Treatment achievements and the risk for relapse vary over time, so the objectives of the treatment plan must account for characteristics of the pre-treatment stage and the availability and capacity of individuals to restructure their social network, besides the opioid maintenance treatment and institutional social care.

## Background

Opioid maintenance treatment (OMT) for opioid dependence (OD) implies using pharmacologic therapies such as buprenorphine or methadone opioid agonists. Psychosocial interventions adjunctive to OMT have proven to improve treatment retention [1]. The scientific literature has consistently reported the association between OMT and outcomes reflecting positively on health administration, social services, and criminal justice, besides the personal benefit for those suffering from an OD. Direct results of OMT, such as the reduction of opioid use, overdose deaths rates, treatment dropouts, number of new HIV infections due to injection risk behaviors, drug-related criminal activity, successful elimination of hepatitis C, and an overall increase in the quality of life of those affected by OD have been found in many studies [2–10]. Despite the repeated empirical demonstration of OMT’s positive outcomes, the treatment mechanisms that make a sustained recovery possible are not well understood yet, and in that regard, this treatment is occasionally categorized as a “black box” [11]. The term “recovery” has been used with different meanings. For example, the Betty Ford Institute Consensus Panel [12] has defined it as “a voluntarily maintained lifestyle characterized by sobriety, personal health, and citizenship” (p. 222). Likewise, the UK Drug Policy Commission [13] conceives recovery as a process over time in which the individual attains control over substance use which allows participation in the roles and responsibilities of society and maximizes health and well being. On the other hand, Best and colleagues [14] assessed recovery experiences among individuals enrolled in drug treatment services in the UK and found that the use of some psychoactive substances was not inconsistent with a recovery journey for some individuals. In this study, the expression “sustained recovery” indicates the maintained abstinence of non-prescribed opioids (NPO) and other non-prescribed psychoactive substances (NPPS).

Treatment success, defined as the achievement of sustained recovery and an increase in the level of the individuals wellbeing, most likely depends on a combination of patient, therapeutic, and program factors [15] working in an unknown process. For persons with problematic use of NPO, easy access to opioid medication and appropriate dosage have been demonstrated as critical factors [16, 17]. However, the value of other treatment components, such as urine monitoring, counseling, psychotherapy, psychiatric care, and the provision of social assistance, has been questioned, although there is evidence that different approaches, including those involving cognitive behavioral models and mindfulness-based techniques have a positive impact on relapse prevention [18, 19]. Amato et al. [20] compared 27 quantitative studies that analyzed the benefit of diverse types of intervention and psychosocial support with the results of OMT, using indicators such as retention in treatment, abstinence of NPO, psychiatric symptoms, and treatment compliance. Contrary to expectations, this meta-analysis failed to find significant differences between interventions that implied distinct treatment components, and none of them stood out for their high efficacy. Based on such evidence, there is a risk that treatment policies that advocate for facilitating access to opiate substitution prescriptions while limiting or eliminating adjunctive psychological and social interventions [21] might have unforeseeable consequences.

Besides the lack of insight into treatment mechanisms, the definition of “successful treatment” is also controversial. Sustained recovery is a slow and difficult process for most people with OD [22]. Outcomes of OMT vary enormously, and retention in treatment seems to be more an exception than the rule [23]. High rates of relapse and infrequent long-term abstinence of NPO have caused some authors to classify OD as a chronic disorder [22, 24]. Factors such as low medication dosage level, lifestyles that complicate medication management, and problematic interactions between patients and program staff reportedly lead to treatment drop out [25–28]. High frequency of opioid use prior to the initiation of OMT and sociostructural factors such as low income and unemployment are also related to attrition [29, 30], mainly in countries with significant social inequalities [31] where impoverished populations lack access to private insurance, for example [32].

The chronic nature of OD has driven the opinion that OMT should have an open-end structure and be continued lifelong when needed [33]. Consistent with this approach, usage of the life-course framework [34] aids in understanding which factors related to OMT contribute to long-term abstinence of NPO. The life-course paradigm highlights the sequence of roles and social transitions occurring during an individual’s life [35]. Following this paradigm, treatment might be considered a turning point [36] or a change in the life trajectory for those with OD. Turning points can occur in two ways: as abrupt ruptures in the life course, or, more in accordance with the usual pattern of desistance of NPO, through repeated cycles of use-treatment-relapse, as a process over time. A turning point separates past from future in the individual’s history, contrasting life under substance misuse with recovery to social functioning, self-improvement, and a generally healthier lifestyle. But what psychological mechanisms motivate individuals to abstain from problematic use of NPO, and what motivates them to sustain recovery? Two theoretical models of motivation can help us answer this question. First, the theory of planned behavior [37] postulates that attitudes, subjective norms, and perceived behavioral control determine the individual’s intention to perform a behavior and the intention, together with the perception of control of their conduct, significantly explains the actual behavior. *Attitudes* refer to the degree of preference for or against a behavior. *Subjective norms* refer to the level of perceived social pressure to execute a behavior or not. Finally, *perceived behavioral control* refers to the judgment the individual makes about the degree of ease or difficulty of effectively performing the behavior. Applying these principles to OMT for OD, we postulate that an individual’s intention to desist from opioid intake should depend on their attitudes toward terminating problematic use of NPO and eventually other NPPS, the perceived social pressure to cease their substance use habit, and their appraisal of their ability to do so. Second, Maslow’s motivational model [38] postulates that human behavior can be attributed to the necessity to satisfy needs in five domains: physiological, safety, love and belonging, esteem, and self-actualization. Maslow initially proposed his model as a hierarchy, with physiological needs at the bottom and self-actualization on top, where the needs further down the hierarchy must be satisfied before individuals can attend to the needs higher up [39]. However, in a later version of his work, Maslow acknowledged that most behavior is multi-motivated, and the order of needs might vary depending on external circumstances and individual differences [40].

Sweden’s drug-treatment-dominant position is abstinence-oriented and based on a non-medical social model [41, 42]. It is a mirror image of Swedish drug policy in general, in which the vision of a drug-free society has shaped the aim that drug treatment should also lead to total abstinence [43]. A recent study comparing four Nordic countries found that access to care for those with OD in Sweden is more limited than in the other three countries [44]. Although methadone maintenance was introduced in Sweden in the early 60s, it was long considered strictly as an experimental method and discarded as a primary alternative for the treatment of OD [45]. Today, the predominant public perception of opioid agonist therapies in Sweden is still that patients are substituting one drug for another. This misconception engenders prejudice and discrimination [46, 47] and, to a certain extent, forces patients to conceal their status.

OMT in Sweden is highly regulated and restrictive, reflecting a model that has been described as high-threshold and low-tolerance [48]. *High-threshold* refers to structural barriers, such as the requirement that a specialist in psychiatry certifies that OD has existed for at least 12 months prior to entry into treatment and a minimum age of 20 years, with exceptions for special cases [49]. *Low-tolerance* refers to the regulations and policies that patients must obey while in treatment, such as the obligation of repeated drug testing and not consuming any type of non-prescribed drug. Needless to say, the low-tolerance component of the model has been associated with high rates of attrition [48]. In Sweden, OMT patients are formally enrolled in health care, but with additional control mechanisms not required in general medical care. Until 2016, besides the requirement of drug testing, patients were likely to be dismissed from treatment if they fail to follow the individual care plan [50, 51]. In 2016, discharged rules were officially removed by a new regulation [49], but controlling measures are still seen as necessary for patient safety and to minimize the risk that medically prescribed opioids are leaked to the illegal market [52]. The new regulation is less prescriptive and leave it to the discretion of the medical professional in charge to establish: (1) the treatment plan, (2) medical checks to be performed during treatment, and (3) special conditions that apply to treatment. Before OMT starts, patients must complete an initial evaluation to establish the severity of their dependence, whether the patient presents a substance use disorder for alcohol or other psychoactive substances, and whether starting OMT might present a hazard for the patient’s health if other substance use disorders are present [49]. An individual care plan is then established. This plan includes an initial medication dosage, calibrated afterward based on patient tolerance, psychiatric assistance if deemed necessary, voluntary individual or group sessions for relapse prevention, and support regarding the social situation, housing, and employment if needed [49].

We conducted this study in the Swedish context with a twofold objective: First, to analyze which factors related to OMT for OD contribute to the abstinence of NPO and sustain recovery from the individuals’ perspective; second, to understand which changes OMT produced in the individuals’ lives might significantly contribute to relapse prevention.

## Method

### Study design

We employed a cross-sectional design and convenience sampling. We collected the data using qualitative methods of inquiry, aiming to obtain different shades and details of the phenomenon under analysis and using an inductive approach to the data [53, 54]. We purposefully designed a semi-structured interview containing three parts. First, we started the interview with general questions about the participant’s background (e.g., age, place of upbringing, familial relationships, and past and current employment status), which were useful to achieve a certain level of rapport besides the information we gathered. Afterward, we proceeded to inquire about the substance use career (i.e., first use, escalation, problematic use patterns of NPO and other NPPS, previous treatments). Finally, the third and larger part focused specifically on the OMT, with questions about the motives for choosing this type of treatment, initial moments in treatment, changes in the participants’ lives while under treatment, perceived strengths and weaknesses of OMT, and the perception of how others think about opioid agonist therapies. The regional ethical review committee approved this study.

### Participants

All 118 patients enrolled in OMT for OD in a Swedish region of almost 250,000 inhabitants in 2018 were considered potential participants for the study. Contact and recruitment took place during January and February 2018 through the only clinic in the region providing this type of treatment. The clinic operates in the facilities of a hospital and works similarly to other hospital wards. It employs medical administrators, psychiatrists, psychologists, counselors, and nurses with special training in psychiatry. The patients’ initial assessment, the start of treatment, follow-ups, and urine and medical controls take place in the clinic. During the first three months of treatment, the patients visit the clinic daily to take the medication under the supervision of healthcare professionals. After three months, patients who do not test positive for other psychoactive substances beyond the treatment are eligible to take the medication home for 2–3 days or a week. Long-term patients who keep testing negative are eligible to collect the medication in pharmacies and have less frequent controls. Besides the opioid maintenance medication, patients’ receive psychiatric assistance, supportive conversations, telephone counseling and are offered voluntary group sessions of cognitive-behavioral relapse prevention. The relapse prevention takes place once per week and lasts for an average of eight weeks.

Deficient understanding or expression of the Swedish language was the exclusion criterion for our study, which none of the initially contacted patients presented. Not all 118 patients had equal opportunity to participate in the study because recruitment occurred while the patients visited the clinic for treatment, which occurred with different frequencies, depending on the treatment stage. Patients visited the clinic daily, every two days, weekly, or monthly and some even acquired their medication in pharmacies without the necessity of visiting the clinic. A research assistant or clinical staff first approached the individuals in the waiting room, provided an information sheet, and briefly introduced the study’s objectives. The research assistant provided further information about the research and conditions for participation (i.e., voluntarism and confidentiality) to those patients who showed interest (25 of 30). During the two months of the recruitment process, 19 patients agreed to participate in the interviews. Characteristics of the participants are displayed in Table 1.

**Table 1:** Characteristics of the study participants

Characteristics	Description
Sex	
Male	*n = 16 (84.0 %)*
Age	
Md; IQ_R_; Rank	40; 5.75; [28 – 53]
Currently employed	
Yes	*n = 3 (16.0 %)*
Age of first use of NPPS	Between 12 and 15
Length of current treatment	
Less than 12 months	*n* = 4 (21.1 %)
12–24 months	*n* = 6 (31.6 %)
More than 24 months	*n* = 9 (47.4 %)
Type of medication	
Methadone	*n* = 6 (31.6 %)
Buprenorphine	*n* = 13 (68.4 %)
Types of previous treatments^a^	
Abstinence-oriented	12-Step; Narcotics Anonymous; religious support groups; psychotherapy; detoxification + psychotherapy
OMT	Methadone maintenance; buprenorphine maintenance
Frequency of clinic visits^b^	Every second day; every third day; weekly; biweekly

^a^All participants reported having been in at least one type of treatment before the current OMT program; 3 participants reported previous OMT experiences; 17 participants reported having tried abstinence without any support.

^b^ Participants were given medication to take home for one week or every 2 weeks, but they also visited the clinic for analytical checks, counselling, psychotherapy, and relapse prevention sessions, or just for social relations.

Initially, the objective was to reproduce in the sample the 30 % rate of females in treatment in the region in 2018, but women more frequently declined to participate. Those who declined to participate alleged they could not stay for the duration of the interview due to different motives (e.g., felt sick, had a tough treatment session at the clinic, lived far away and needed to arrange transport, time shortage due to other reasons). The researchers obtained the participants’ consent prior to conducting the interviews, which took place one-on-one at the clinic in a designated room to ensure privacy and confidentiality. The participants were informed that the researchers had no contractual relationship with the clinic, that no information would be collected from their clinical files, and that the clinical staff would not have access to the information participants disclose during the interview. The interviews were conducted by a female research assistant, with an academic degree in criminology, who had received training for qualitative research interviewing and specifically for conducting the study’s interviews.

After each interview, the authors reviewed the data to ensure that the method yielded the information necessary to address the objectives and that code and meaning saturation was achieved [55]. Code saturation for both objectives was achieved first around interview 12, but the researchers decided to continue the interviews to ensure meaning saturation. Finally, when the 19th patient was interviewed, it was corroborated that no new codes, categories, or themes were found, and the concepts of the theory developed in the analysis of the second objective were well developed, as advised by Morse [56].

All participants indicated having previous work experience, although only three were employed at the time of the interview. The participants’ age of first drug use varied between 12 and 15 years old. All participants reported a history of using multiple NPPS, but the use of heroin or other opioids had been most prevalent in their lives before they started OMT. The length of time in the current treatment varied; one participant had started treatment only a few months before the interview, while another participant reported having started 18 years prior.

### Data and analysis

The information analyzed included self-reported data provided during the one-on-one interviews. The interviews were 15 to 50 min in length, recorded and transcribed afterward, and anonymized by code assignment. A pseudonym was assigned to each code to facilitate reading through the results section.

The analyses were performed in two steps serving each of the objectives. The first step included content analysis, according to the five phases proposed by Yin [54], namely: summary, dismantling, remounting, interpretation, and conclusions, to answer the question, “What determines the abstinence of problematic use of NPO and sustained recovery?” The transcribed material was read repeatedly to search for patterns and disassemble data into codes following an inductive approach [57]. The remounting phase revealed a structure that clearly differentiated between factors determinant of success occurring during the pre-treatment and the treatment stages. Because the length of time in treatment significantly varied between participants, the transcripts of those who had been in treatment for a longer time (i.e., 18 or more months) without interruptions were initially analyzed separately. After all, they could be considered as being in sustained recovery, while the same cannot be considered for the group of patients with a shorter enrollment period (i.e., less than 18 months). We decided to take this approach even though there is no way to determine whether someone will take drugs again in their lifetime. This consideration is only probabilistic, meaning that those not taking NPPS for a longer period are less likely to relapse than those who have recently abandoned a substance use career [36]. We decided to independently analyze both groups, searching for differences in the discourses of the motivation to maintain abstinence from NPO and eventually other NPPS. Because the analytical categories found in the transcripts of both groups converged, we decided to report all the participants together in the Results section.

During the second step of the analysis, we used a grounded theory approach [58, 59] to answer the question, “What existential changes does OMT promote that might contribute to relapse prevention?” We developed a coding scheme to categorize common themes and elaborated patterns and linkages between categories, carrying out constant comparisons between codes (i.e., fragments of the data), concepts (i.e., significance assigned to a code) and categories (i.e., concepts of higher order) as suggested by Strauss and Corbin [60]. The initial coding scheme emerged after iterative reading of the first five transcripts and evolved through its application to the other 14 transcripts. Afterward, we retrieved and analyzed content from all the transcripts by code to further understand and refine the categories and achieve common themes. In the end, three themes were obtained, which further conceptually framed the findings of the first step.

The authors decided to use two different analytical approaches because while the first objective had a preconceptual framework based on Ajzen’s theory of planned behavior [37], and was used therefore as a deductive approach to structuring the results, there was no initial theorizing when approaching the second objective. Regarding the second objective, the authors were interested in understanding the underlying processes, and built the theory by testing hypotheses generated through critically reading the empirical data in a complete inductive approach. Maslow’s theory of needs [38] surfaced after the analysis as the best explanation to frame the results.

Both authors participated in the entire process of analysis. The authors have translated all quotations from Swedish into English.

## Results

**Fig. 1:**
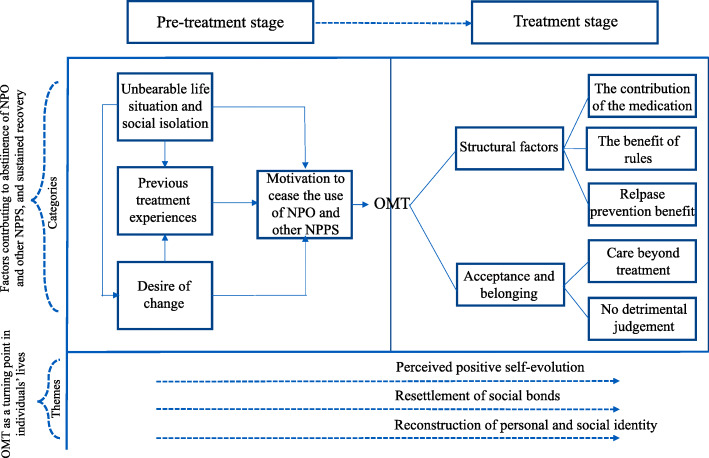
Diagram of categories and themes found during data analysis. The upper diagram plots categories and their relationships found during content analysis. At the bottom is displayed the themes produced during the grounded theory analysis

We organized categories and themes found during data analysis in a flow diagram, plotted in Fig. 1.

After an initial period of experimenting with drugs during adolescence, all participants in our study evolved through cycles of substance use, treatment, desistance, recovery, and relapse before starting the current OMT program (i.e., pre-treatment stage). Internal (i.e., cognitive and affective) processes, the social environment, and different events that occurred in the participants’ lives during the pre-treatment stage motivated them to abstain from drug intake, while the treatment motivated them to sustain recovery. Two opposing driving forces were buried deep inside the participants during their substance use career and at the initial stages of treatment. On one side, there was a desire to make lifestyle changes, enhanced by the vision of returning to a functional life, while on the other side, the drug cravings were constantly pulling them back. By a *functional life*, we mean the ability to secure housing and employment and to reestablish severed ties with family and friends. The participants perceived that under the current OMT, a transformation started to occur. The transformation that produced the eagerness to sustain recovery was mainly related to the satisfaction of certain psychological needs such as safety, love and belonging, or esteem postulated by Maslow’s motivational theory [38–40]. The participants did not perceive OMT as an isolated event in their lives, but rather a process occurring within a specific social context. They deemed the structural factors and the sense of acceptance and belonging as essential to supporting the transformation. Additionally, the social context and the perceived warmth displayed by the clinical professionals were considered of great importance for treatment success.

From the participants’ perspective, OMT promoted a positive self-evolution, the possibility to resettle social bonds, and, subsequently, the reconstruction of a new personal and social identity. The level at which this transformation is attained might contribute to determining the recovery or, conversely, future relapse. We analyzed the factors that promoted abstinence from problematic use of NPO and other NPPS and the perspective of treatment as a turning point separately.

### Factors contributing to abstinence of NPO and other NPPS and sustained recovery

There were circumstances in the participants’ lives before they started the current OMT, which progressively strengthened their motivation to stop abusing drugs and seek treatment.

#### Pre‐treatment stage

Before they entered the current OMT, the participants’ daily lives were dominated by substance misuse and their struggle to find the resources to nurture their dependence. The participants reported that individuals with an OD are never satiated. Although opioids provided many pleasant feelings and sensations, the withdrawal symptoms and unpleasant emotions produced if drugs were not available dominated the individual’s inner lives and pushed them to an almost continuous search for more drugs and resources to buy them. During their substance use careers, there were moments when the participants perceived their lifestyle as almost unbearable and impossible to continue for long. “Kevin” explained:

You wake up in the morning and you feel really bad, so you have to get hold of money to buy it [heroin]. Then you have to walk around and steal, or commit a burglary... often [I] was shoplifting during the day... and you sell it [the stolen merchandise] to get money, then you get drugs, then you take it, then it started all over again. A fucking squirrel wheel.

As the participants kept using NPO, they started to develop many physical and psychological symptoms, which created a significant strain that became difficult to manage. They revealed that they did not perceive that they were in control of their behavior and had developed feelings of helplessness. At the same time, the participants hid their substance misuse from those in their immediate environment who could have ultimately served as social support. Overwhelming feelings of guilt and shame dominated their emotional life, leading them to sever all bonds with relatives and friends. “Matts” decided to leave home when he started taking NPPS:

I wouldn’t care about having a good life, I just wanted to feel good, but I wouldn’t ever expose my mother to the turmoil and the torments ... she has been so worried about me, and I feel bad about that.

Isolation from primary groups – a source of love, caring, concern, support, etc. – and reference groups – composed of people who occupy the social role to which the individual aspires –was a problem identified by all the participants during the period in which they were using NPO and other NPPS. A bidirectional causal relationship resulted wherein the participants turned away from family and friends because they were taking drugs, and then they took drugs because they could not bear the feeling of loneliness. Besides those with whom the participants shared the drug-related environment, they became more secluded, trapped in a progressively more unsustainable lifestyle. The loss of jobs and other normative sources of income, the lack of resources to maintain a high level of NPPS consumption, and the absence of social support all contributed to their psychological strain. They informed us how social isolation carves hard within the individual not only while using NPO and other NPPS, but is also carried like a heavy burden when enrolling in treatment. “Markus” pointed out:

The first thing I did was to break with everyone. I threw away the phone for a whole year. I think that was what saved me... you get a little lonely when you become drug-free, at the beginning.

Their unbearable life situation and the social isolation led the participants to an increasingly strong desire to modify their lifestyle to achieve a certain level of normative social functioning. All participants described initial use NPPS during early adolescence, mainly with alcohol and cannabis, with a quick escalation to using other substances such as opioids. They reported that when dependency struck, the desire for change grew stronger, reinforcing positive attitudes toward treatment and furthering the motivation or intention to quit using NPO and other NPPS. In some cases, an event that had significant meaning for the individual triggered or reinforced the desire for change. In the case of “David,” it was the illness of his mother:

When my mom was lying in bed at the hospital I thought I had to do something about it [substance misuse]; she just can’t die knowing that I, yes, I’m doing it [taking drugs]

As the motivation to cease taking NPO and other NPPS grew, the participants highlighted that they started seeking treatment. All the individuals in our study disclosed having been involved in treatment several times before starting the current OMT. Some of them perceived the experience of recurring cycles of substance misuse-treatment-relapse as a personal failure, contributing to the feeling that abstinence was not under their control. “Robert” informed us:

I have tried many treatments... In the end, I just felt that I can’t bear it anymore, [to] begin treatment after treatment.

The mechanisms triggered by the treatment that made sustaining recovery possible seem complex. All the participants had tried different types of abstinence-oriented programs, which they perceived as riskier for relapse than OMT. In effect, some of the participants never achieved total abstinence while under other types of treatment, such as the 12-Step program. While the peculiar characteristics of OMT generally seem to increase the motivation to cease NPO and NPPS intake completely, some participants reported relapsing after previous OMT experiences. The medication was not enough if an internal conversion was not achieved. “Per Olof,” who had started the current treatment five months earlier, had tried OMT for the first time a year before the interview but eventually relapsed. He informed us:

I went into the program, I received the medication (...) I had not been there before. I thought with the medication everything would be solved magically, but it was not so. I still felt bad inside [...] I had methadone, changed to buprenorphine... It was only a waste of time.

Some participants reported taking the same substance(s) that doctors prescribed to them for treatment purposes (i.e., buprenorphine) before they started the current OMT as their main misuse substance because it was more readily available and cheaper in the illegal drug market than other types of opioids. However, before they started treatment, they were unable to achieve all the changes that treatment facilitated because they had adopted a criminal lifestyle to find resources to buy the drugs. While the level of motivation or the intention to stop using NPO and other NPPS might have eventually varied from one individual to another, a certain level seems necessary to sustain recovery. In this regard, “Peter” told us:

It is not possible to turn off just because you get enrolled [into treatment]. You still have it [the dependency]. You get a small dose to help cope with it [withdrawal symptoms], but yes. I mean, just because you get in [treatment] you aren’t clean. It’s a daily work. You work every day with yourself to stay clean.

In sum, the participants in our study reported how the life situation and the desire for change added up to a certain level of motivation that shaped their intention to cease the use of NPO and other NPPS and drove them to seek treatment. Sustaining recovery depends partially on factors related to treatment and partially on achieving a sense of acceptance and belonging to primary and reference groups.

#### Treatment stage

The participants deemed the medication essential in reducing the physical symptoms of withdrawal and also the anxiety produced by the mere idea of feeling the symptoms if opioids were not available. The medication reduced drug cravings, facilitating that the participants were not concentrating on obtaining drugs most of their time. In this way, the participants perceived an enhancement in their psychological wellbeing. “Elias” explained:

The dose I have keeps me healthy around the clock... and that helps psychologically too.

However, OMT’s power to keep participants away from NPO use lay not only in the medication. Besides the physical dependency, OD seems to imply certain psychological effects from the participants’ perspective that are important to consider during treatment. “David” referred to the cognitive and affective processes necessary to complement the medication:

[Treatment] is about working with yourself. The medication is just a small part of the treatment itself. It’s not that you come here and take the medication and then life is OK. It doesn’t work like that.

All the participants in our study emphasized the importance of social interaction, critical because, as we saw earlier, social isolation was nearly always present in the individuals’ lives during their substance use career and at the beginning of treatment. “Johanna” stated:

The medication is just a small piece of what you get here because here there are people you can talk to...

The participants regarded the rules to continue treatment established by the clinic, such as the prohibition of using any type of NPO and other NPPS and compulsory urine testing as hard, but necessary and positive for treatment success. However, to be willing to accept the rules, the individuals thought it was important they were equally applied to everyone and displayed in a context of warm relationships with the program staff. It was important that the participants not perceive the staff as guards, but rather as friendly professionals. The participants highly valued the relapse prevention sessions. The cognitive-behavioral therapy strategies taught during the sessions helped the participants change negative thinking and develop coping skills. Learning such strategies provided the individuals with important tools to manage not only their opioid dependency but, more generally, the addictive behavior. “Kevin” explained:

After four sessions [in the relapse prevention program] things started to happen within me. After 10 weeks... I have totally redone my way of thinking... quit taking a lot of medicines. I try not to find my happiness in chemicals anymore.

Besides any new knowledge the participants may have acquired during the relapse prevention sessions, they perceived the repetitive practice of identifying the clues that trigger the drugs cravings and the rehearsal of behavioral strategies to handle these tense situations until achieving a certain level of automatic response, as having a therapeutic effect.

Interestingly, OMT has other components than the content of the treatment that the participants perceived as fundamental to its success. These other components are related to the feeling of acceptance and belonging, very close to the essential human psychological needs that act as motivators of behavior. The participants valued the current OMT because the professionals in the clinic covered these needs for them in some way, solving the problem of social isolation built under a relatively lengthy substance use career. From the individuals’ perspective, it was not only about social interaction, but also about feeling that someone cared and was concerned, and about finding attachment figures among the clinical professionals and eventually among other patients of the OMT program. The quality of the relationships established between the clinical staff and the participants, beyond the strictly professional requirements, ultimately produced feelings of care and acceptance similar to those we find in primary social groups like the family. “Johanna” explained:

Here, there are people you can talk to, people who work here, who listen to you, and understand why you feel like you do, and that is of great value. One must be able to talk to someone without being treated like an idiot.

The participants experienced strong stigmatization not only during their substance use career but also when entering treatment. The individuals perceived that they were continuously subjected to detrimental judgment in many social situations. Furthermore, previous treatment experiences might have contributed to generalizations about the treatment setting and the feeling of constantly being judged. Finding a group in which the individual felt accepted as it has occurred in the current OMT program reinforced the motivation to secure the place in such environment. The social climate that the participants in our study experienced in the OMT clinic was in clear contrast with what they had experienced in the past in other environments, including other OMT scenarios. “Kevin” informed us:

They [the clinical staff] see me as a human being and not as an addict... I have experienced it over the years like, yes, people looking down on me.

In short, during the treatment stage, there were factors directly related to the OMT characteristics such as the medication, the rules, and the relapse prevention sessions that the participants perceived as necessary for sustaining recovery. However, the warm relationships that the participants established with the clinical staff and the sense of belonging to a social group in which they felt they were accepted independently of their life course were valued as highly as the treatment in preventing relapse.

### OMT as a turning point in individuals’ lives

The second level of analysis, to determine what existential changes participants perceived were fostered by the OMT, revealed three themes.

The first theme was “Perceived positive self-evolution.” The participants thought about themselves as if they were walking a path to achieve a constructive personal existence. They described a process of change that they believed would make possible what they most yearned for, a functional life. The individuals viewed OMT as a lock mechanism that opened the doors to this path, and they thought they must cross it by themselves. “Johanna” declared:

I think that the program is a damn good thing... I want a productive life... and I want a healthy life... and if you want, they will gladly help you.

To a certain extent, the participants reported feeling they were responsible for their lives again, in contrast with their previously perceived lack of control. Participants referred to a new lifestyle that clearly broke from their lifestyle while using NPPS. In this sense, they described how OMT represented that point in the life course when aspects that could be classified as socially disadvantaged or even antisocial changed to socially accepted. Some of the older participants had experienced this before in their multiple experiences of abstinence and rehabilitation. However, we found that they had the same idealized expectations about the future as the younger participants who had never been fully employed or had never lived independently from their parents. They were not anticipating the burdens that a prosocial lifestyle entails, perhaps because they were just too jaded about them while using NPO and other NPPS.

. “Leif” stated:

The goal is that you have to come out to work, yes, get a new life, or get an apartment, get out to work. That’s what I see is the most important right now.

However, the individuals recognized that the process of change was not easy and that it would take time. Due to their past experiences with other treatment programs and relapse, participants were convinced that the achievements that OMT facilitates required effort from them and that it would not be easy to deal with the negative emotionality that had been easily relieved through drug intake in the past. Especially during the first months of treatment, the participants reported cycles of mood swings that could destabilize their motivation to sustain recovery. “Markus” revealed:

You have been doing drugs for 10–15 years. It’s not easy to quit just like that. There is a period when you are up and down.

In this process of change, participants had to deal with mechanisms of positive reinforcement to maintain abstinence that were delayed in time, were occasionally not immediately evident for them, and occasionally alternated with negative reinforcement by the environment and by undesirable psychological strain and physical pain. This was the opposite of the immediate reinforcement the individuals obtained when taking drugs. “Mats” revealed:

It really depends on you. You have to come to an insight into what you want in life.

Therefore, learning what triggers drug cravings and impulse control (i.e., components of relapse prevention sessions) was imperative for them. Moreover, the lack of social skills and stigmatization was a doubly disadvantaged starting point for the process of self-evolution, which also required learning and training. “Markus” reported:

It’s a damn break from how you used to live. You have to learn new things. The worst is, after all, this social part, as well as coming out again [socially] in a new way. I still have a hard time talking to people. It takes time, everything.

OMT boosted personal growth. The individuals reported they had a new sense of achievement and dignity that came from a certain sense of self-fulfillment and that they could eventually perceive respect from others. The participants who had been under the current OMT program for a longer time had established a clear difference between how well they felt in general with themselves compared to how they felt while using NPO and other NPPS. “Markus” related:

I’m feeling good. When I got into this [treatment]... I’m not thinking about the drugs, I don’t have to worry about the aches anymore. So, yes, I’ve got a whole new life. For me it is. And you don’t want to get rid of that.

Even those participants who had started OMT more recently, such as Fredrik, described this positive self-evolution:

I have been coming here for a year and this year has been so good. Yes, probably I’d never had better years... It’s different [from the previous life while taking drugs] like night and day.

Although OD has been seen as a chronic health problem, and some individuals might require OMT permanently, many of the participants in our study reported that their goal was to reach a functional life, free of medication. They depicted an inner feeling of freedom and the realization of personal potential. “Per Olof” recounted:

I have a dream that sometime in my life, I can wake up one day without having to take pills. But I’m not going to rush, but I’m building it up.

A second theme found during the analysis of OMT as a turning point was the “resettlement of social bonds.” Most participants in our study had severed bonds with their families at one time or another during their substance use career. While some informed us that their families “gave up” on them, others decided to hide their substance use and cut relations unilaterally to prevent family members from suffering. Upon starting treatment, they viewed the resettlement of these bonds as a primary necessity. Beyond the feelings of love and belonging, the family represented a means of establishing an environment where the individuals felt safe and secure and experienced acceptance, order, and control over their lives. In sum, familial relationships created a social comfort zone. “Eva” reported:

For me, the family is a support in my life because they help to continue to recover and not go into drugs again. Yes, they help me both mentally and physically. It is a support for me anyway.

However, the individuals were susceptible to how family members perceived and felt about them. The quality of the relationship had been severely affected by the substance misuse for most of the participants, and at the beginning, family members were suspicious of the individuals’ behavior. On the other hand, they were susceptible to family behaviors that they ultimately perceived as dismissive. However, the continuation in OMT facilitated the reinstatement of trust and confidence, and the participants informed us that it was of great relief and joy when they finally achieved them. Only then was it possible to construct truly supportive relationships. “Joseph” reported:

It’s great [the family relationship] right now, now that things have gone well for so long. They started to trust me now that everything starts to work well [because of the treatment]. They are very happy... It became a completely different relationship. If you take drugs, it’s not possible to have any relationship, so it’s a huge difference.

Besides the family, the participants highly valued the warm therapeutic relationship established with the OMT clinical staff. Some participants, who had been in OMT before in other clinics and who did not, for whatever reason, develop the same kind of relationship, pointed it out as an adjuvant factor for treatment success. When re-establishing bonds with the family was not possible, the clinical staff functioned as a substitute for the primary social group. Communication with the clinical professionals and a warm affective climate was deemed so important that some participants decided to continue treatment in the clinic even when they were eligible to access their medication through the pharmacy distribution net. “Mats” said:

I like them [the clinical staff]. It’s very nice to meet them. It’s people who take part in me, in my well-being, and how I feel, and that makes me like to come here to get the medication.

While the reinstatement of relationships with primary social groups was deemed fundamental, establishing relationships with peers and peripheral social groups differed depending on individual preferences. Most of the participants informed us that they enjoyed making relationships with other patients in the clinic who helped them construct a sense of inclusiveness. “Leif” told us:

I think it’s fun to just sit and talk with everyone here while taking the medication... also with the staff.

In comparison, “Markus,” who had been in treatment at the clinic for one and a half years at the time of the interview, preferred to stay away from other patients because he identified them as a risk factor for relapse:

I don’t hang out with anyone that comes here... don’t want to get dragged into any fucking shit. I have to keep that distance for myself... Often those who come here they talk only about drugs and it’s not so fucking fun. That’s what I’m trying to get away from. I don’t hang out with anyone, just with the kids, mother, dad, brother, sister...

For some, establishing bonds with others was challenging, and despite all the other components of treatment, unattended feelings of isolation and loneliness could remain, which individuals perceived as a threat of relapse. “Dan” revealed:

The biggest problem is that you don’t have any friends. Then it is normal that you turn to your old friends... It is very difficult as an adult to get new friends, which is probably the biggest problem.

In this sense, the availability of potential affective sources and the capacity of the individual to establish social bonds should be evaluated and prioritized structurally during the treatment, and it should not be left to chance for individuals to manage these necessities on their own.

The third theme found when analyzing OMT as a turning point was the “reconstruction of personal and social identity.” Individuals who abstained from taking NPO and other NPPS and endured the recovery referred to themselves as completely different people compared to when substance misuse dominated their lives. The sense of self-fulfillment and social functioning, and the new lifestyle created a new identity. The participants reportedly replaced the “hooked on opioids person,” as they used to see themselves, with a friendlier and more pleasant person, which was a matter of pride for them. “Anders” reported:

[I went] from being a junkie who walked around the street and maybe scared people to sitting in town and talk to any lady or old man. It’s a huge difference.

The new identity contained aspects related to the realization of personal potential, including parenthood, successful marital relationships, and success in the workplace. “Kevin” reported:

Now I have a partner, two children, a permanent job. Yes, life works like life should work... great.

However, because the misconception persists that OMT is about “state-provided drugs”, the individuals constantly struggled with the new identity they were trying to construct and the image of an active drug user, as society classified and labeled them. OMT may be a turning point for the individuals, but not so for others in society. “Estelle” told us:

It feels like people don’t like it... I know people who think we are drug abusers, that we are not drug-free. So it’s terrible, terrible.

The participants informed us that opposing forces against OMT transpired not only from the general public but also from specific social groups. They indicated, for example, that advocates of abstinence-oriented treatments shared the stigmatization bias. “Maria” reported:

There are people who have the opinion that it [medication] is a drug from the state. Also, the 12-Step movement thinks like that. Many people think we come here because we get drugs for free.

They also perceived stigmatization from other social groups considered “deviant.” “Per Olof” revealed:

There is a motorcycle club that is alcohol and drug free. I am not welcome there because I take medication and they think I am an addict then.

Despite their struggle against stigma, the participants in our study perceived they were finding a place in society, facilitated by their new identity. They felt as if they achieved a status like other sick people who need treatment. According to “Adam”:

It’s just like any disease. If you have a blood disease or something, then you have to take medication. It’s the same here [with OMT].

## Discussion

In this study, our objective was to look inside the “black box” of OMT for OD through the patients’ eyes to understand what components contribute to a sustained recovery from substance misuse and to understand which changes this type of treatment produced in the individuals’ lives might significantly contribute to relapse prevention.

We assumed there were factors related to treatment success prior to OMT [29, 30]. We found that the motivation to abstain from problematic use of NPO and eventually other NPPS during the pre-treatment stage that moved the individuals to seek OMT worked jointly with factors occurring during the treatment stage to explain treatment outcomes. The motivation to quit NPO use seems to build upon the three factors postulated by the theory of planned behavior [37]. First, a favorable attitude toward the abstinence of NPO and eventually other NPPS appears to have originated in an unbearable life situation and the social isolation that individuals with OD find themselves in at one point or another during their substance use career. This supports the idea that individuals with substance use disorders seek treatment not as an end per se, but rather as a means of escaping negative consequences and improving their quality of life [61, 62]. Second, we found that the stigma felt by the individuals while they were using drugs was difficult to manage, consistent with previous empirical findings [46, 63, 64], and it worked as a perceived social pressure to increase the motivation to quit using NPO and other NPPS. Third, increasing the level of self-control played an important role in remaining abstinent, as was found in previous studies [65]. The positive expectations of self-efficacy or perceived control to effectively quit using NPO produced by earlier experiences of treatment and relapse also contribute to enhancing the motivation to get into OMT, which reinforces the role of self-efficacy as a predictor and mediator of treatment outcomes found in quantitative studies [65–67].

In short, when individuals arrived at OMT, they carried a certain level of motivation to abstain from using NPO and other NPPS that contributed to determining what would happen inside the black box of treatment and, subsequently, the treatment outcomes. Therefore, the pre-treatment stage should be seen as an area to evaluate and consider when establishing the individual treatment plan.

Contrary to findings in several quantitative studies [20], individuals perceive relapse prevention sessions and other components of the individual treatment plan as important as the medication dosage during the treatment stage. The cognitive-behavioral therapy strategies taught during the sessions helped participants change negative thinking and develop coping skills, just as they are intended to work [68]. This is a finding to consider for any potential policy advocating for delivering medication without accessory costs [21], mainly in countries where psychological treatment or psychosocial support is not prescribed in the regulations as part of OMT.

Moreover, the individuals reported undergoing the internal psychological processes of maturation and personal growth like those described in previous research [69, 70], and perceived a change in their life course consistent with Elder’s conceptualization of turning points [34]. Reading these results in the context of Maslow’s motivational theory [39], the different OMT components facilitate the satisfaction of human needs at several levels and are a key to helping patients gain a sense of normalcy [71]. At the most basic level, it is necessary to consider that OD has a neurobiological basis [72]. While the medication works to cover basic physiological needs otherwise disturbed by withdrawal symptoms, the support regarding the social situation included in the individual plan covers the needs related to safety, security, and living resources. Because support networks have been found to play a crucial role in sustaining recovery [73, 74], they should be considered when the individual starts treatment. On a superior level of the needs hierarchy, the social climate of the OMT clinic, and the quality of the relationships the individuals were able to establish with the clinical professionals and other patients of the clinic, as well as the reinstatement of familial bonds covered the necessities of love and belonging. Building upon the lower levels of the pyramid of needs, the individuals were then able to deploy personal resources to work on their inner needs of respect, self-esteem, and recognition. The increase in self-regulation and self-efficacy was related to remaining abstinent [66, 75], most likely because the perception of behavioral control was enhanced in a feedback cycle that further promoted the motivation to sustain recovery. In this sense, the meaning of sustained recovery included general aspects of wellbeing and was holistic, bringing it closer to the definition agreed upon by the Betty Ford Institute Consensus Panel [12] and the process defined by the UK Drug Policy Commission [13].

It is necessary to keep in mind that individuals reach a point of fulfillment of their necessities at particular moments during their lives, depending on individual differences. Therefore, treatment achievements, as well as risk for relapse, vary in time, and the objectives of the treatment plan must account for these individual differences. As Best and colleagues [76] pointed out, patients’ assessment generally concentrates on most urgent needs, but for a treatment journey to be effective, it is necessary to proceed comprehensively and evaluate the different levels within the hierarchy of needs model. While for some individuals, the risk of relapse may start to decrease immediately upon starting treatment, others will need more time until this occurs, and it is possible that, for some, the risk of relapse will remain at a relatively high level.

 From the participants’ point of view, OMT opened the door to certain existential changes, which they perceived as a positive self-evolution. Individuals walk a path from deviant patterns of conduct to a normative lifestyle. Furthermore, OMT potentiates the resettlement of social bonds and the reconstruction of personal and social identity. OMT is indeed a turning point that can only be understood through a life-course perspective [77]. During their substance use career, the individuals experienced personal identities that they avoided meeting in the mirror. They departed from a point devoid of existential meaning in the sense that it was the opposite of what Maslow [39] proposed as self-actualization, the realization of a person’s full potential and personal growth. OMT allowed the trajectory to run in the opposite direction, although the participants experienced strong stigmatizations when entering treatment because OMT in Sweden is generally perceived as a “drugs provided by the state” program. Whether the individuals take this other direction or not depends on them, but opioid substitute medication, social care, favorable conditions for developing a sense of belonging, and reinstatement of bonds with primary and reference social groups are essential factors.

The results of this study should be considered in light of some limitations. First, the study was limited to one point in time. Because we applied the life-course retroactively, we do not know the trajectory of the individuals after we interviewed them for whether they sustained recovery.

Second, the study used convenience sampling, which does not guarantee generalization of the results to all patients under OMT in the unit or in the Swedish context. Furthermore, the low number of female participants did not allow for an analysis of potential gender differences. Third, in 2018, in the Swedish region where the study took place, patients in OMT were still stigmatized because it was not abstinence-oriented, and the clinic followed a high-threshold/low-tolerance model.

Further research should study individuals longitudinally and include participants who identify with genders other than male. Reproducing the study with different OMT models of threshold and tolerance and in social contexts where non-abstinence-oriented treatments are more accepted is necessary to generalize the findings.

## Conclusions

Besides individual psychological differences, OMT for OD outcomes depends on multiple factors occurring in pre-treatment and treatment stages that must be considered when establishing an individual treatment plan. Motivation to cease the intake of NPS and eventually other NPPS builds upon social isolation, unbearable life situations and previous treatment experiences that enhance the desire for changing and eventually bring individuals to seek OMT.

The quality of the human relationships that individuals are able to establish with a supportive social network, including professionals in the treatment setting, is as important as structural treatment factors to sustain recovery and should not be left to chance for individuals to manage on their own.

OMT may represent a turning point in individuals’ lives as long as it allows them to resettle the social bonds, walk a path perceived as a positive self-evolution, and reconstruct a personal and social identity that contrasts with the identity they acquired during their substance use career.

## Data Availability

The data analyzed during the current study are available from the corresponding author upon reasonable request.

## Abbreviations

OMT: Opioid Maintenance Treatment
OD: Opioid Dependence
NPO: Non-Prescribed Opioids
NPPS: Non-Prescribed Psychoactive Substances
